# Functional analysis of BmTsp.C in modulating infection of BmNPV through apoptosis pathways in domestic silkworm (Bombyx mori)

**DOI:** 10.1099/jgv.0.002098

**Published:** 2025-05-09

**Authors:** Yuanyuan Xu, Yimeng Wei, Jing Zhang, Dan Zhang, Qiaoling Zhao, Dongxu Shen

**Affiliations:** 1 Jiangsu Key Laboratory of Sericultural and Animal Biotechnology, School of Biotechnology, Jiangsu University of Science and Technology, Zhenjiang 212100, PR China; 2 Key Laboratory of Silkworm and Mulberry Genetic Improvement, Ministry of Agriculture and Rural Affairs, Sericultural Scientific Research Center, Chinese Academy of Agricultural Sciences, Zhenjiang 212100, PR China

**Keywords:** apoptosis, BmNPV, bombyx mori, tetraspanins

## Abstract

The silkworm, *Bombyx mori*, is a crucial model insect in agriculture and biological research. Tetraspanins, known for their effects in regulating cellular functions like cell signalling, adhesion, migration and diffusion, take on a crucial role in viral dynamics, influencing both viral spread and entry into host cells. In this study, a tetraspanin gene called *BmTsp.C* from the silkworm genome was identified and investigated. Tissue profiles showed that *BmTsp.C* has the highest transcription level in midgut, with a marked increase following viral infection. The immunofluorescence localization suggested that BmTsp.C is primarily distributed on the cell membrane. Additionally, overexpression of *BmTsp.C* in BmN cells facilitated the proliferation of BmNPV. Meanwhile, siRNA-mediated knockdown of *BmTsp.C* could inhibit viral proliferation. In addition, knockdown of *BmTsp*.C at the individual level further validated the remarkable effect of *BmTsp.C* during viral infestation. Furthermore, overexpression of *BmTsp.C* could regulate the expression of apoptosis-related genes. Results from flow cytometry indicated a decrease in the number of apoptotic cells after overexpression of *BmTsp.C*. Taken together, our results demonstrated that *BmTsp.C*, as an important factor in the Tetraspanin-enriched microdomains, exerts a significant influence on the proliferation of BmNPV, most likely through the cellular apoptosis pathway.

## Introduction

The silkworm (*Bombyx mori*) (genus*: Bombycidae*; order: Lepidoptera: family: *Bombycidae*) is an economically significant insect and a crucial model organism in biological and genetic studies [1]. The *Bombyx mori* nucleopolyhedrovirus (BmNPV) is a typical pathogenic baculovirus that causes severe viral diseases in sericulture, which can bring about substantial economic losses [2]. Therefore, it is necessary to elucidate the resistance mechanisms of silkworms against BmNPV [3].

Understanding the molecular mechanisms of BmNPV infection and the silkworm’s immune response is crucial for relieving viral damage. A central immune response in silkworms is apoptosis, which is also a defence mechanism in the immune response or when cells are invaded by harmful substances and plays a role in the elimination of infected cells [4,5]. In addition, apoptosis involves morphological and biochemical changes including cell shrinkage, nuclear condensation, DNA breaks and formation of apoptotic bodies. These apoptotic bodies are subsequently phagocytosed by neighbouring cells or immune cells, thereby preventing the release of harmful cellular contents [6]. Virus-induced apoptosis serves a dual role in the complex interaction between host and pathogen. Cells infected by viruses can trigger apoptosis as a defensive mechanism to eliminate the infected cells and impede the progression of the viral infection. Conversely, viruses can exploit apoptosis to facilitate their release and subsequent spread [7,9]. As for BmNPV, after infecting BmN cells, it can affect the apoptotic pathway through various mechanisms, thus enhancing its own replication and proliferation. For example, the *p35* gene has been shown to block caspase activity, effectively inhibiting apoptosis and ensuring the stability of the viral proliferation environment [10]. In addition, the *Bombyx mori inhibitor of apoptosis (Bmiap*) gene inhibited apoptosis in *Bombyx mori* cells and promoted the proliferation of BmNPV [11]. Furthermore, LINC5438, which is related to apoptosis in *Bombyx mori*, was found to promote BmNPV proliferation and effectively inhibit apoptosis in cells when overexpressed.

In the process of apoptosis, the death signal is transmitted through a complex signalling pathway and involves a series of signal transduction events [12]. Apoptosis is a process that involves the liberation of cytochrome C (cyt C), the activation of caspase enzymes and the involvement of various regulatory proteins such as Bcl2, culminating in cellular mortality [13]. Eventually, cells undergoing apoptosis give rise to apoptotic bodies, which undergo phagocytosis [14]. In contrast, cells that are unable to proceed through apoptosis may lead to the accumulation of damaged cells within the organism, heightening the risk of oncogenesis [15]. Regarding silkworm cells, the release of cyt C from the mitochondria into the cytoplasm is a crucial step in initiating apoptosis, triggered by external stimuli. The cytoplasmic cyt C associates with Apaf1, facilitating the activation of Caspase 9. This activation acts as a catalyst for the triggering of subsequent caspases, thereby initiating programmed cellular demise [16].

Tetraspanins are integral components of a large family of proteins characterized by four transmembrane (TM) domains, two extracellular loops (LEL and SEL) and one intracellular loop, which have been extensively studied due to their roles in creating intricate networks on the cell membrane and diverse functions [17]. Research suggests that tetraspanins have a significant impact on facilitating the entry of viruses into target cells by gathering host factors and forming complexes with related viral membrane proteins [18]. Several tetraspanins act as pre-viral aggregators of host factors, while others form complexes with related viral membrane proteins for endocytosis [19].

Tetraspanin-enriched microdomains (TEMs) are formed by tetraspanins and interact with various membrane proteins, including integrins, growth factor receptors and immune receptors [16]. Additionally, TEMs can bind to viral structural proteins, promoting viral budding and membrane fusion for viral particle release [20]. For enveloped RNA viruses such as Coronaviruses (CoV) and Influenza A viruses, TEMs containing CD9 and CD81 molecules have been identified as critical entry sites required for viral fusion [18]. In arthropod hosts, tetraspanins play a significant role in viral transmission. Studies have indicated that *Culicidae* (genus: *Diptera*; order: Diptera: family: *Culicidae*) Tsp29Fb mediates the transmission of Dengue virus from mosquito to mammalian cells [21]. Moreover, tetraspanins interact with various membrane proteins and intracellular signalling molecules, serving as a pivotal part in cell signalling, adhesion, migration, spreading and aggregation of cell surface receptors [22]. In *Drosophila* (genus: *Diptera*; order: Diptera: family: *Drosophilidae*) immune cells, Dfos, a member of the fruit fly proto-oncogenes (Fos), regulates TM4SF mRNA levels and affects macrophage mobilizing and invading the embryonic germ band. Furthermore, TM4SF forms networks with actin on the surface of macrophages and promotes the activation of diaphanous protein, which supports macrophage mobility to the intra- and extracellular interface [23].

In recent years, the connection between tetraspanins and apoptosis has become increasingly apparent. For instance, CD82, a mammalian tetraspanin, has been identified as a crucial regulator of apoptosis, particularly through its influence on the PI3K/Akt signalling pathway. A decrease in CD82 expression correlates with an increase in PI3K/Akt pathway activity, resulting in significant inhibition in apoptosis, which is observed in certain cancers [24]. Human Tspan1 is frequently overexpressed in a variety of tumour cells. Reducing its expression can inhibit the migration and invasion of human pancreatic cancer cells [25].

In this study, a tetraspanin gene called *BmTsp.C* was identified from the silkworm transcriptome database [26]. Preliminary bioinformatics analysis indicated that BmTsp.C has typical tetraspanin characteristics, which may play a crucial role in viral infections. Expression profiles showed that *BmTsp.C* has the highest transcription level in midgut and is dramatically induced upon BmNPV infection, and the expression level of *VP39* further confirmed it. Immunofluorescence localization studies suggested that it was mainly distributed on the cell membrane. Furthermore, the overexpression and knockdown assays conducted further demonstrated that *BmTsp.C* was able to enhance BmNPV infection by modulating the expression of apoptosis-related genes. In addition, the knockdown in silkworm bodies suggested that *BmTsp.C* has a significant effect in midgut and further validated the effect of *BmTsp.C* in apoptosis pathways both *in vivo* and *in vitro* models. Furthermore, flow cytometry results also indicated that *BmTsp.C* possesses the ability to inhibit apoptosis. In summary, our results lay the foundation for further elucidation of the molecular function of *BmTsp.C* and the mechanism of BmNPV invasion.

## Methods

### Silkworm cultivation, cell and virus preparation

The *p50* silkworm strain, obtained from Jiangsu University of Science and Technology, Zhenjiang, Jiangsu Province, was reared under controlled conditions (25 °C ± 1 °C, 75% relative humidity) and a 12 h light/dark cycle, with daily fresh mulberry leaves feeding. BmN cells, originating from silkworm ovaries, were cultured in TC-100 medium (Living Biotechnology Technology Co., Ltd., Beijing, China) supplemented with 10% fetal bovine serum (SinoBiological, Beijing, China), 100 µg ml^−1^ penicillin and streptomycin. The BmNPV tagged with EGFP (BV-EGFP), developed and maintained in our lab, was utilized to evaluate viral infection and proliferation.

### Bioinformatics analysis of *BmTsp.C*

The sequence information of *BmTsp.C* was obtained through the NCBI database (https://www.ncbi.nlm.nih.gov/). Molecular weight and isoelectric point predictions were performed by using EXPASY (http://www.expasy.org). Signal peptides were predicted by using SignalP-5.0 (https://services.healthtech.dtu.dk/services/SignalP-5.0/). The topology of the protein was analysed by using Protter (https://wlab.ethz.ch/protter/start/). Multiple sequence alignment analysis was carried out by using BioEdit software. The analysis of homologous sequences was used by the blast tool in NCBI, and the phylogenetic tree of BmTsp.C was constructed by using mega7.0. Furthermore, the subcellular localization was predicted by Cell-PLoc 2.0 (http://www.csbio.sjtu.edu.cn/bioinf/Cell-PLoc-2/).

### Vector construction and siRNA synthesis

Specific primers containing restriction sites *Kpn* I and *Xba* I were designed to clone the ORF region of *BmTsp.C*. The PCR product was ligated into the pMD-19T vector for sequencing verification. Then, the PCR fragment was cloned into the pIZT/V5-His vector for recombinant vector construction, confirmed by sequencing at Shanghai Sangon Biotech (Shanghai, China). Additionally, specific *siRNA* targeting *BmTsp.C* was synthesized by GenePharma (Suzhou, China), with the *siTsp.C* sequence as treated group (5′-GGAGUGGCCGUCUUAAGAATT-3′), and *siNC* sequence as control group (5′-UUCUCCGAACGUGUCACGUTT-3′).

### Transient transfection, viral infection and fluorescence analysis

BmN cells were placed in 12-well cell culture plates and left to attach to the wall, and overexpression plasmids or siRNA were transfected into BmN cells (60%–70% confluency) using GP-transfect-Mate reagent (GenePharma, Suzhou, China). After 24 h of overexpression, the cells were infected with BmNPV-EGFP with a multiplicity of infection (MOI) of 3. An inverted fluorescence microscopy (Ti-E, Nikon, Japan) was used to observe the fluorescence distribution of BmNPV in cells at 24, 48 and 72 h post-infection.

### Knockdown in silkworm bodies

Ten silkworms of the same size and condition on the third day of the fifth-instar larva of strain p50 were selected and injected with siNC and siTsp.C, siNC as the control group and siTsp.C as the experimental group. Each silkworm was injected with 3 µg of siRNA, and after 4 h of injection, they were fed with mulberry leaves containing wild-type BmNPV of 1.0×10^7^ OB ml^−1^ (occlusion body), and then after the silkworms consumed the BmNPV-containing leaves, an equal amount of clean mulberry leaves was fed to them. The midgut of silkworms was extracted after 48 h of injection.

### RNA extraction and cDNA synthesis

The third day of the fifth-instar larva of strain *p50* was stunned on ice, and larvae were dissected to collect tissues including the head, fat body, hemolymph, midgut, silk gland and Malpighian tubules. These larvae were fed with mulberry leaves soaked with BmNPV suspension (1.0×10^7^ OB ml^−1^), and tissues were collected at 24, 48 and 72 h post-infection. Total RNA was extracted from these tissues by using Trizol (Takara, Tokyo, Japan) and preserved immediately in a −80℃ freezer. First, we took samples from the freezer and put them in ice to carry out follow-up operations. Next, 200 µl chloroform was added per millilitre of Trizol, mixed every tube and then centrifuged them at 12,000 rpm for 15 min at 4 ℃ to precipitate protein. Then, collected supernatant into new tubes and added equal volume of isopropanol. Whereafter, stand at room temperature for 10 min and centrifuge samples under the same conditions for 25 min. Finally, washed them twice with 75% ethanol and waited for blow dry, dissolved the precipitate by using DEPC water. RNA purity and concentration were ascertained by using a NanoDrop 2000 spectrophotometer (Thermo Fisher Scientific, New York, USA). The reverse transcription was performed by the FastKing RT Kit (Tiangen Biotech, Beijing, China) to synthesize cDNA, and the procedure was in accordance with this instruction.

### Genomic DNA extraction

Following collection of BmN cells infected by BV-EGFP at different times and subjected to DNA extraction. Briefly, an appropriate amount of buffer (10 mM Tris-HCl, 100 mM EDTA, 100 mM NaCl, 0.5% SDS, pH = 8.0) was added to the collected samples and ground, followed by an equal volume of saturated phenol (pH = 7.4). After thorough mixing and centrifugation at 12,000 rpm for 15 min, the supernatant was transferred to new tubes. The solution consisted of chloroform:isoamyl alcohol (3 : 1) was added, followed by anhydrous ethanol and NaAC (3M) to precipitate DNA at −20 °C. The precipitate was then centrifuged and washed twice with 75% ethanol, then dissolved in DEPC-treated water and stored at −20 °C for subsequent experiments.

### Quantitative real-time PCR analysis

The BmN cells were collected after overexpression of knockdown at different time points. And, RNA or genomic DNA was obtained by the methods described above. The transcription levels of templates were measured by quantitative real-time PCR (qRT-PCR) assay by using UltraSYBR mixture (Comwin Biotech Co., Ltd., Beijing, China) on a LightCycler 96 PCR instrument (Roche, Basel, Switzerland). The amplification by initial denaturation at 95 °C for 15 min, followed by denaturation at 95 °C for 15 s and annealing at 60 °C for 30 s, for 40 cycles. All experiments were repeated independently three times with biological replicates, and each sample was tested in duplicate for technical replicates. The data analysis was done by using the 2^−ΔΔCt^ method to calculate relative mRNA expression or genomic DNA copy numbers with *BmGAPDH* as an internal reference.

### Cellular apoptosis analysis and morphological observation post-overexpression

Cells were infected with BV-EGFP in the experiment of transfecting BmN cells with *BmTsp*.C overexpression vector, and cells transfected with the empty vector were used as control. After 48 h infection, the medium was removed and cells were fixed with 4% paraformaldehyde solution for 10 min, followed by three washes with sterile PBS. The cells were then stained with 0.5 µg ml^−1^ of 4,6-diamidino-2-phenylindole (DAPI) (Sangon Biotech, Shanghai, China) for 15 min in the dark. Finally, the cells were then observed and recorded under an inverted fluorescence microscope.

### Annexin V-FITC/PI apoptosis and flow analysis

Briefly, the BmN cells were transfected by pIZT/V5-His-BmTsp.C and then infected with BmNPV at a MOI of 3, and cells transfected with pIZT/V5-His vector and infected with BmNPV were used as control. Then, cells were rinsed three times with sterile PBS after 48 h post-infection and labelled with staining the Annexin V-FITC and PI apoptosis detection kit (Beyotime, Shanghai, China), according to the procedure. The labelled cells were analysed by using a flow cytometer (Becton, Dickinson and Company, New Jersey, USA) with data processed by FlowJo 10 software.

### Immunofluorescence analysis of BmTsp.C

In experiments where BmN cells were overexpressed by *BmTsp.C*, which were placed on cell creeps for 24 h. The medium was removed and the cells were washed three times on a shaker with sterile PBS for 3 min each time. Cells were fixed with 4% paraformaldehyde solution for 20 min. Subsequently, cells were permeabilized with 0.5% Triton X-100 for 20 min, followed by three washes with PBS. Next, cells were blocked with 5% BSA blocking solution for 1 h, and washed three times with PBS, and then incubated overnight at 4 °C with a mouse monoclonal antibody against polyhistidine (1 : 800, diluted in 5% BSA). After three washes with PBS, cells were incubated with Alexa Fluor 594-conjugated goat anti-mouse secondary antibody (1 : 500, diluted in 5% BSA) for 2 h in the dark. Then, cells were rinsed three times with PBS in the dark, followed by staining with DAPI (0.5 µg ml^−1^) for 15 min, and washed again three times in the dark with PBS. Finally, the stained cell creeps were fixed on slides and observed under a fluorescent inverted microscope (Nikon, Japan).

### Statistical analysis

Each experimental sample was repeated three times, and data were presented as mean ± standard deviation (±S.D.). Data were analysed and compared by using GraphPad Prism 10 software. Different statistical methods were used for analysis, with unpaired *t*-tests used for comparing differences between two groups, and analysis of variance (ANOVA) used for comparing differences among multiple samples (ns, no significance; *P < 0.05; **P < 0.01; ***P < 0.001; ****P < 0.0001).

## Results

### Analysis of tissue and induced expression patterns of *BmTsp.C*

**Fig. 1.**
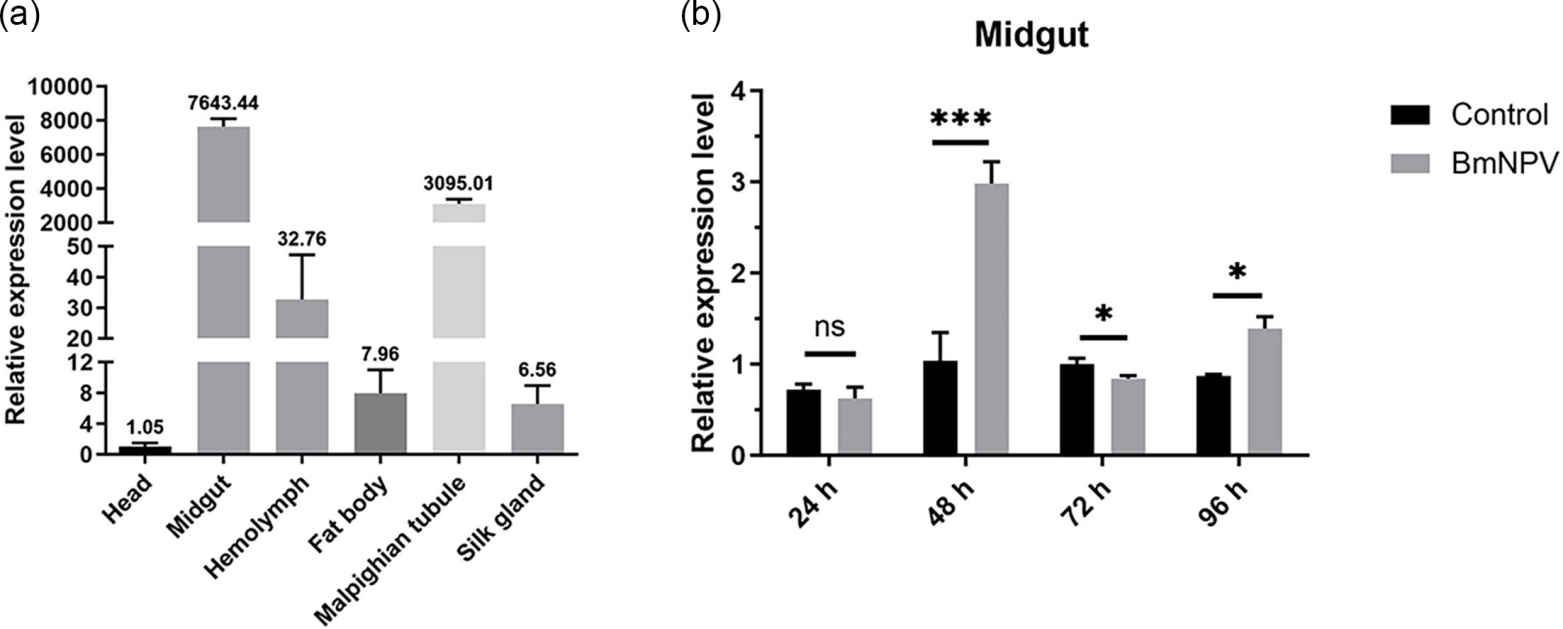
Analysis of tissue and induced expression of *BmTsp.C*. (**a**) The relative expression of *BmTsp.C* in different tissues in silkworm. (**b**) The relative expression of *BmTsp.C* in the midgut at 24, 48, 72 and 96 h. The assay was performed by qRT-PCR with *BmGAPDH* as an internal reference, and data were analysed and plotted by using Prism 10. Error bars represent the mean ± S.D. of three independent values. Asterisks represent the significance of differences between experimental and control groups (unpaired *t*-test; ns, no significance; *P < 0.05; ***P < 0.001).

The relative expression levels of *BmTsp.C* were examined in different tissues on the third day of the fifth-instar by using qRT-PCR. The results indicated that expression of *BmTsp.C* was highest in the midgut, followed by the malpighian tubule and hemolymph, and lowest in the head (Fig. 1a). Furthermore, we assessed the role of *BmTsp.C* upon BmNPV infection by examining its relative expression in the midgut at different time points post-viral infection. Then, we found that there existed dramatic differences in expression levels at 48, 72 and 96 h post-viral infection, which compared to the control group, with the most notable difference observed at 48 h (*P* < 0.001). There was no significant difference in expression levels observed at 24 h post-viral infection compared to the control (Fig. 1b).

### Immunofluorescence localization of analysis of BmTsp.C

**Fig. 2.**
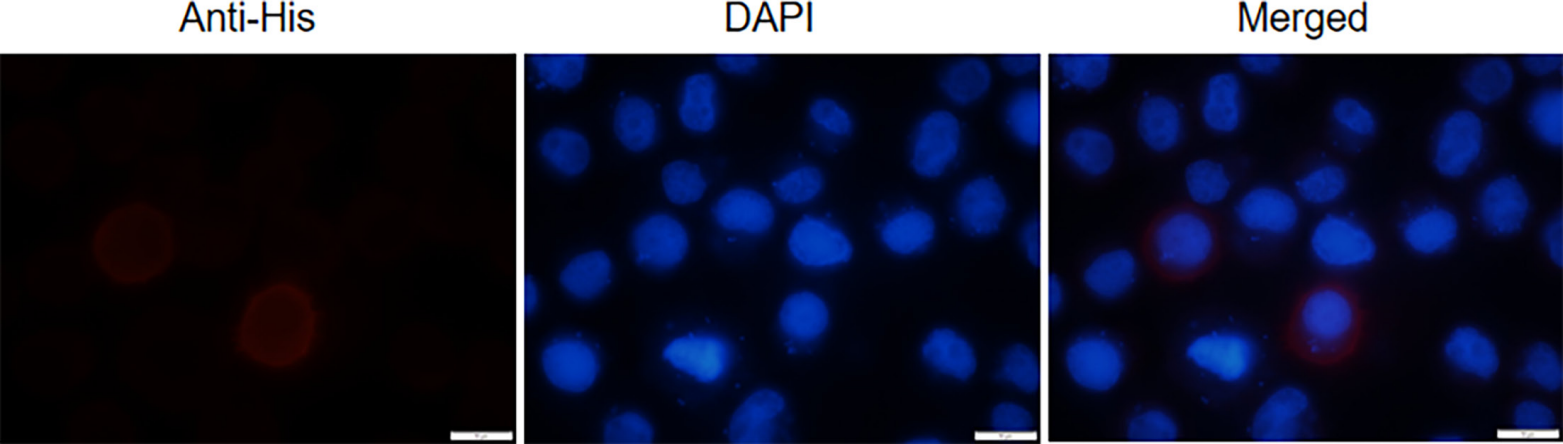
Immunofluorescence localization of BmTsp.C. Cells were transfected with pIZT/V5-His-BmTsp.C plasmid. The fluorescence was seen in an inverted fluorescence microscope at 48 h post-transfection. Alexa-Flour 594-conjugated goat anti-mouse antibody labelled BmTsp.C and was shown in red, and DAPI labelled the nucleus of the cells and was shown in blue. Scale bar = 10 µm.

To determine the cellular localization of BmTsp.C, we embarked on an immunofluorescence localization experiment. Our results revealed that strong red fluorescence was primarily distributed on the cell membrane and was slightly dispersed in the cytoplasmic region, with no significant fluorescence detected around the nucleus (Fig. 2). These observations suggested that BmTsp.C is mainly distributed on the cell membrane, which may be related to several biological processes such as cell signaling transduction and substance transport.

### Overexpression of *BmTsp.C* promotes the infection of BmNPV

**Fig. 3.**
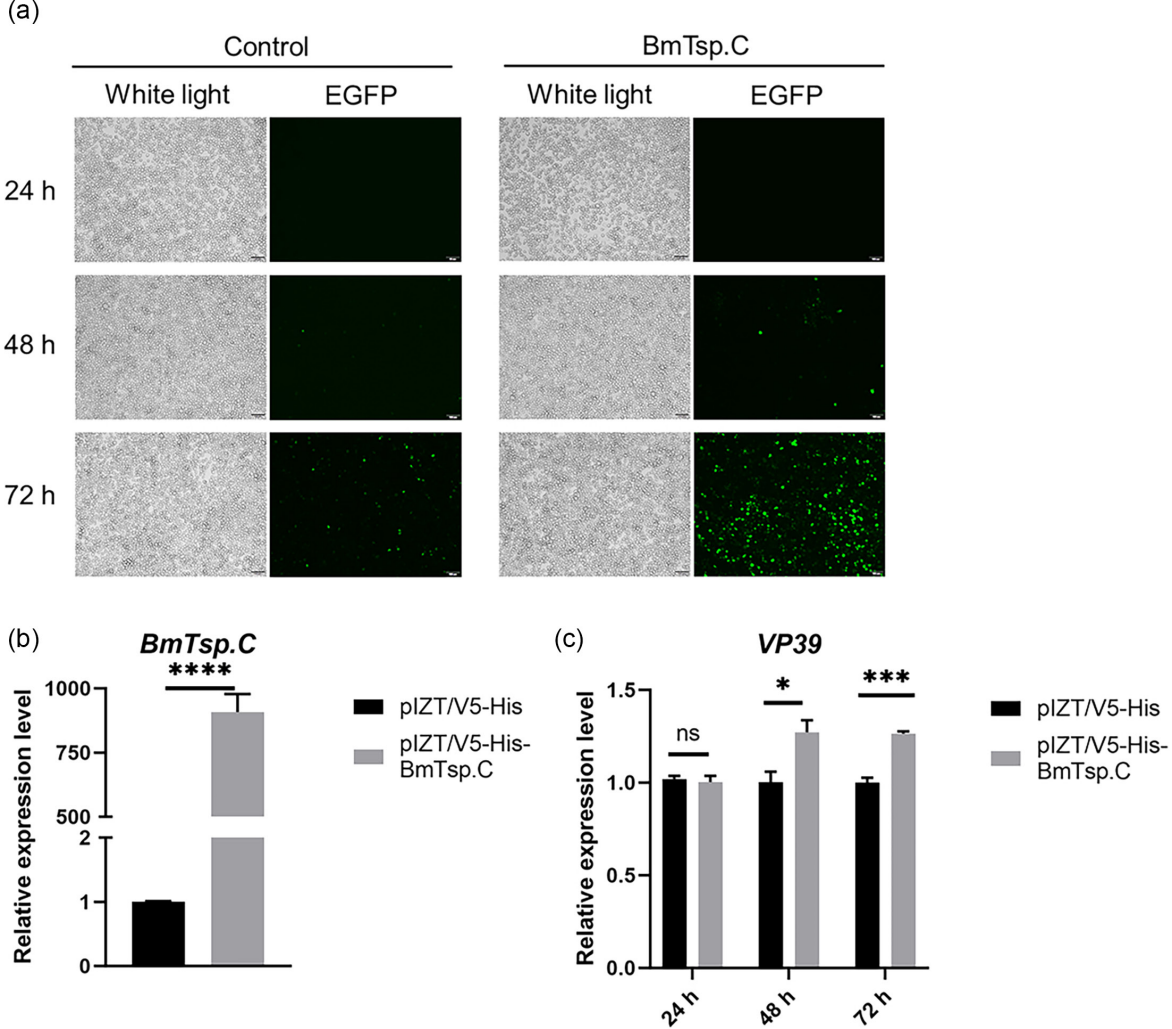
Overexpressed *BmTsp.C* promotes the infection of BmNPV. (**a**) Fluorescence microscopy of BmNPV-infected cells at 24, 48 and 72 h after overexpression of *BmTsp.C*. White light, perspective view; EGFP, green fluorescence. (**b**) Transcription levels of *BmTsp.C* at 48 h after transfection with the overexpression of plasmid. (**c**) Expression levels of *VP39* at 24, 48 and 72 h of viral infection after overexpression of *BmTsp*.C. Samples were analysed by qRT-PCR using *BmGAPDH* as an internal reference, and data were expressed as mean ± S.D. Asterisks indicate the significance of differences between experimental and control groups (unpaired *t*-test; ns, no significance; *P < 0.05; ***P < 0.001; ****P < 0.0001). Scale bar = 100 µm.

To discern the effect of *BmTsp.C* on BmNPV infection, we constructed the pIZT/V5-His-BmTsp.C vector and transfected overexpression plasmids into BmN cells. Notably, a significant upregulation of transcription levels of *BmTsp.C* was observed at 48 h after transfection compared with controls (Fig. 3b). In addition, we monitored the alterations in the expression levels of *VP39* at different time intervals following viral infection by using quantitative qRT-PCR. The data indicated that overexpressed *BmTsp.C* exhibited considerably elevated expression levels of *VP39*, especially at 48 and 72 h upon post-viral infection, compared to the control group (Fig. 3c). Moreover, fluorescence microscopy revealed a prominent increase in green fluorescence intensity in the group with overexpression of *BmTsp.C*, especially compared to the control group, particularly evident at 72 h post-infection (Fig. 3a). Taken together, these results indicated that overexpression of *BmTsp.C* in BmN cells effectively enhanced the infection of BmNPV.

### Overexpression of *BmTsp.C* could regulate the expression of apoptosis-related genes

**Fig. 4.**
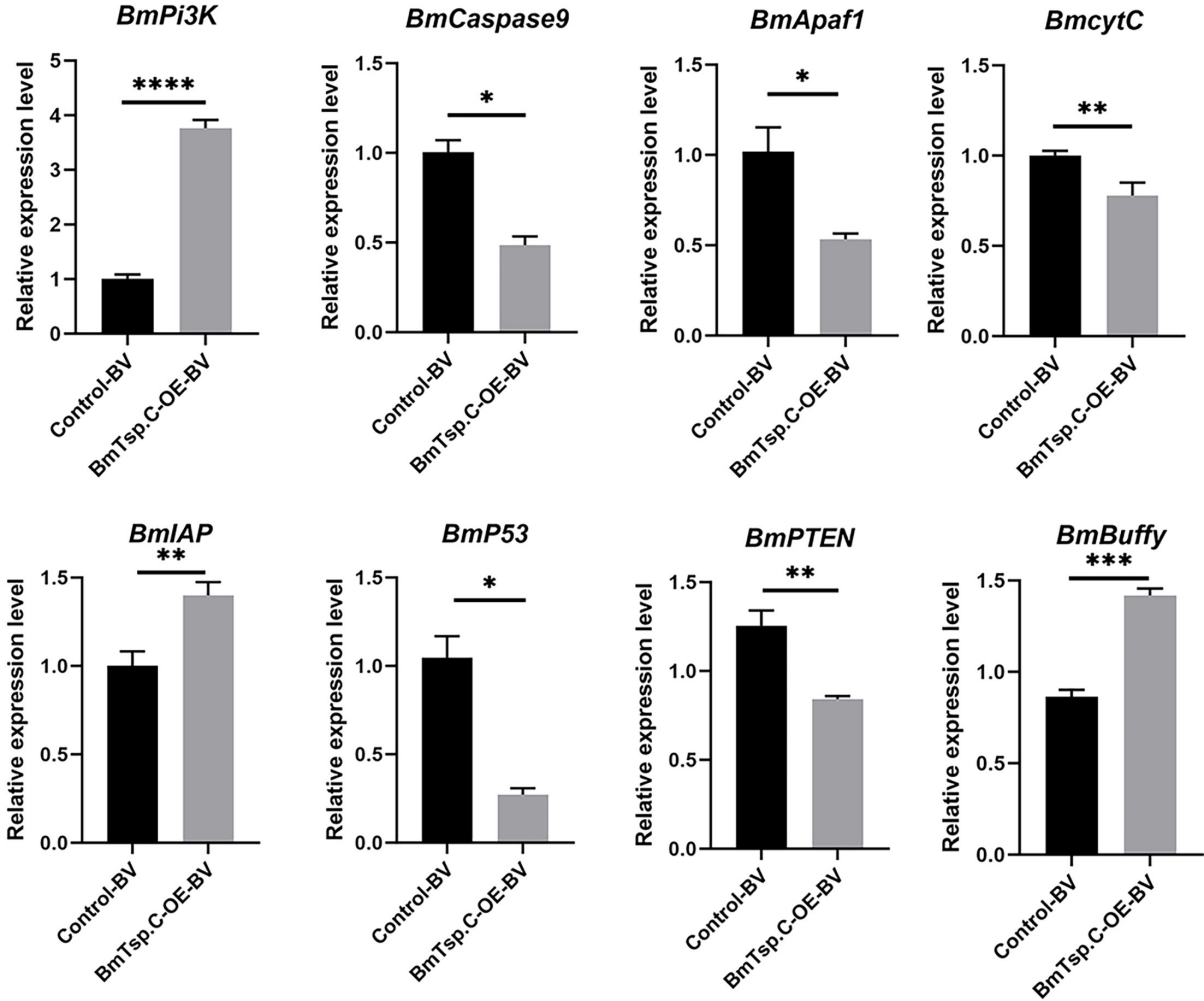
Overexpressed *BmTsp.C* could regulate the expression of apoptosis-related genes. Samples were assayed by using qRT-PCR with *BmGAPDH* as an internal reference, and the data were expressed as mean ± S.D. Asterisks represent the significance of the differences between the experimental and control groups (unpaired *t*-test; *P < 0.05; **P < 0.01; ***P < 0.001; ****P < 0.0001).

To understand whether *BmTsp.C* could affect viral proliferation by modulating apoptosis, we examined the expression levels of apoptosis-related genes in BmN cells transfected with overexpression vector for *BmTsp.C*. After 48 h viral infection, apoptosis-related genes such as *BmPi3K*, *BmIAP* and *BmBuffy* were all exhibited a significant increase in transcription levels. Conversely, genes such as *BmcytC*, *BmApaf1*, *BmCaspase9*, *BmP53* and *BmPTEN* were significantly downregulated in cells in which *BmTsp.C* was overexpressed (Fig. 4). These results suggested that *BmTsp.C* may affect viral infection by orchestrating the expression of apoptosis-related genes in BmN cells.

### Knockdown of *BmTsp.C* has significant effect *in vivo* and *in vitro* models

**Fig. 5.**
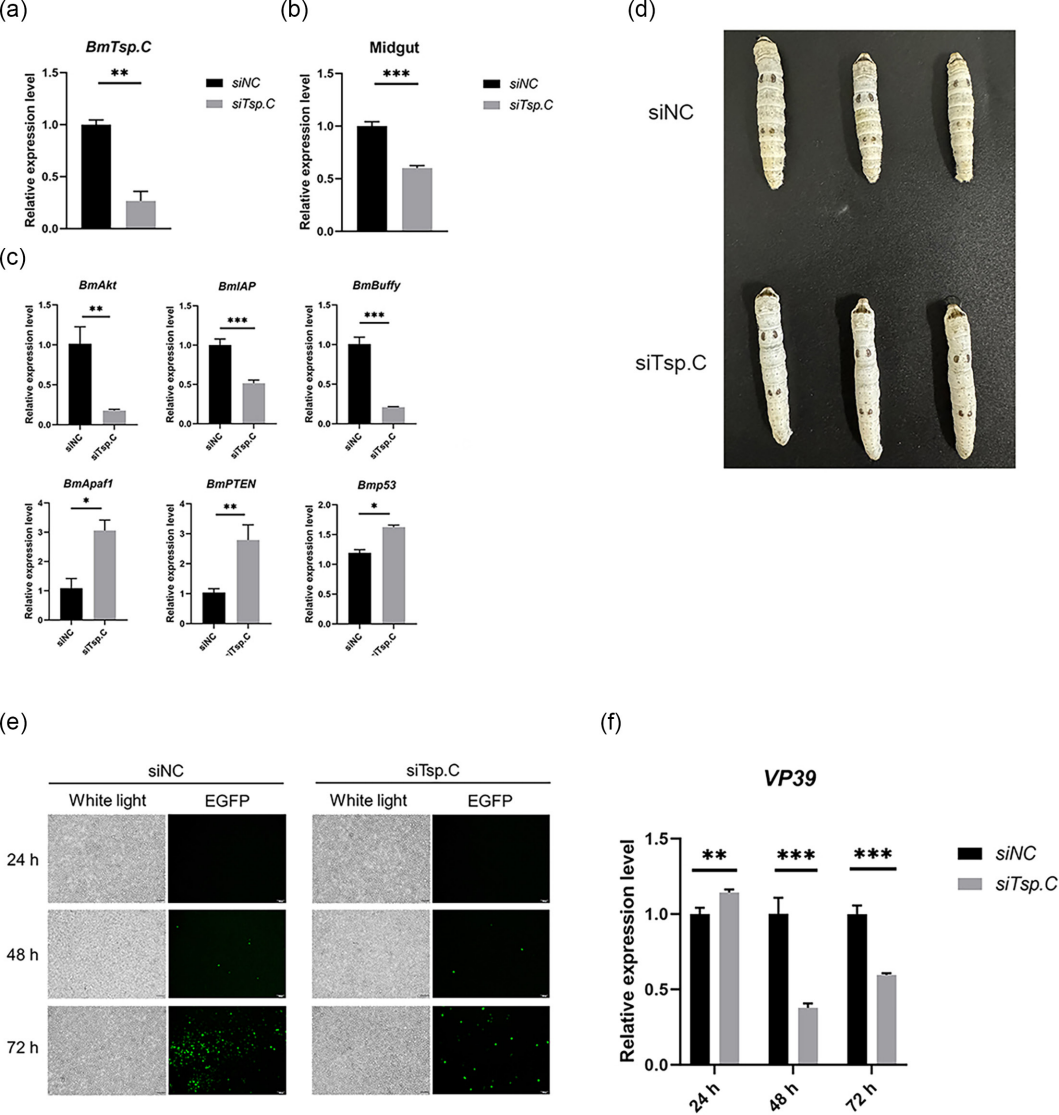
Knockdown of *BmTsp.C* has a significant effect *in vivo* and *in vitro* models. (**a**) Transcription level of *BmTsp.C* at 48 h after transcription of siRNA in BmN cells. (**b**) Transcription level of *BmTsp.C* at 48 h after injection of siRNA in silkworm bodies. (**c**) Expression level of apoptosis genes after knockdown of *BmTsp.C* in silkworm bodies. (**d**) Photos about silkworms 48 h post-injection of siRNA. (**e**) Fluorescence microscopy observation of BmNPV-infected cells at 24, 48 and 72 h after knockdown of *BmTsp.C*. White light, perspective view; EGFP, green fluorescence. (**f**) Expression levels of *VP39* at 24, 48 and 72 h of viral infection after knockdown of *BmTsp.C*. Samples were assayed by using qRT-PCR with *BmGAPDH* as an internal reference, and data were expressed as mean ± S.D. Asterisks represent the significance of the differences between experimental and control groups (unpaired *t*-test; *P < 0.05; **P < 0.01; ***P < 0.001). Scale bar = 100 µm.

To further elucidate the role of *BmTsp.C* in BmNPV infection, we designed siRNA targeting *BmTsp.C* and separately transfected it into BmN cells and injected it into silkworm bodies. In BmN cells, as shown in Fig. 5a, siRNA-mediated knockdown of *BmTsp.C* resulted in a significant reduction in transcription level, and was further corroborated by the knockdown assays *in vivo*, where silkworms injected with siRNA targeting *BmTsp.C* exhibited a significant reduction in *BmTsp.C* expression in the midgut (Fig. 5b). Furthermore, knockdown of *BmTsp.C* in BmN cells can decrease *VP39* expression (Fig. 5f) and fluorescence intensity following BmNPV infection (Fig. 5e), clearly demonstrating that *BmTsp.C* is essential for efficient viral replication at the cellular level. The visual examination revealed that silkworms in the knockdown group remained healthier than the control group. The control group displayed pronounced symptoms of BmNPV infection, including swelling of body segments and milky white body colour, which displayed severe symptoms of BmNPV infection (Fig. 5d). Knockdown of *BmTsp.C* in BmN cells led to reduced viral replication, as seen by decreased *VP39* expression and fluorescence intensity, which is consistent with knockdown in silkworm bodies, where reduced *BmTsp.C* expression in the midgut correlated with healthier larvae and fewer signs of infection. For further investigation of the effect of knockdown of *BmTsp.C* in apoptosis pathways, we inspected the expression level of some important apoptosis genes both *in vivo* and *in vitro*, such as *BmAkt*, *BmIAP* and *BmBuffy*, which were all expressed in reduction, while *BmApaf1*, *BmPTEN* and *Bmp53* showed promotion (Fig. 5c). It is further proven that *BmTsp.C* may affect viral infection according to apoptosis pathways. These findings highlight the importance of *BmTsp.C* in viral proliferation across *in vivo* and *in vitro* models.

### Overexpression of *BmTsp.C* inhibits the apoptosis in BmN cells

**Fig. 6.**
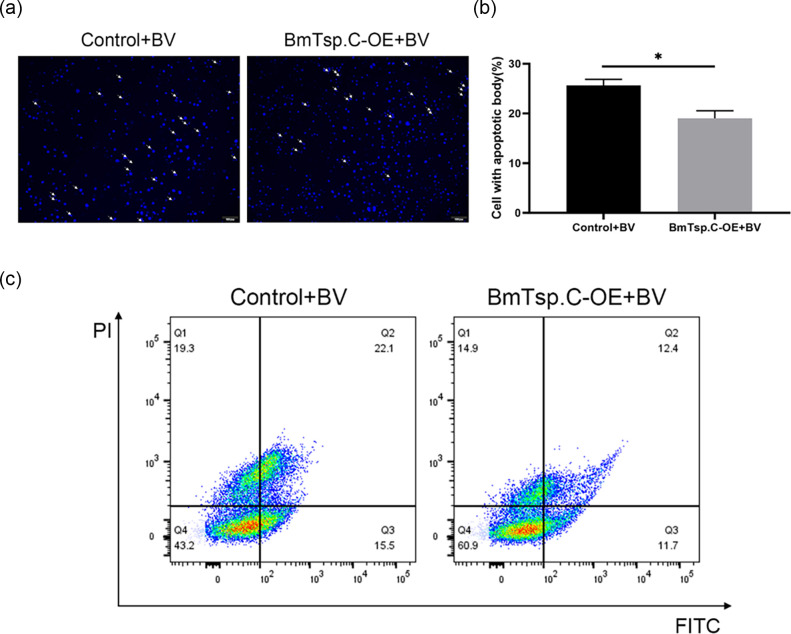
Overexpressed *BmTsp.C* inhibits apoptosis in BmN cells. (**a**) The morphology of the nucleus of BmN cells after overexpressing *BmTsp.C* was observed by using fluorescence microscopy with DAPI staining. (**b**) The percentage of BmN cells showing apoptotic bodies was counted. (**c**) Flow analysis of apoptotic cells was performed by using a scatter plot; Q1 represents early apoptotic cells, Q2 represents late apoptotic cells, Q3 represents normal cells and Q4 represents early apoptotic cells. The scatterplot analysis was obtained by using FlowJo 10. The data were expressed as mean ± S.D. Asterisks indicate significant differences between the experimental and control groups (unpaired *t*-test: *P < 0.05). Scale bar = 100 µm.

To investigate whether *BmTsp.C* promotes viral infection via modulating the apoptosis pathway, we observed and quantified apoptotic bodies of BmN cells overexpressing *BmTsp.C* by using an inverted fluorescence microscope. Compared with the controls, the prevalence of apoptotic bodies in cells with overexpressed *BmTsp.C* was significantly lower after BmNPV infection (Fig. 6a). Moreover, the percentage of apoptotic bodies further substantiated this result (Fig. 6b). Additionally, the status of cell apoptosis was examined through Annexin V-FITC staining. The flow analysis indicated that the proportion of apoptotic cells was reduced to 24.1% in the experimental group, compared to 37.6% in the control group (Fig. 6c), which further represented that overexpression of *BmTsp.C* suppressed apoptosis in BmN cells, compared to the control group. These findings supported the hypothesis that overexpression of *BmTsp.C* in BmN cells could effectively inhibit apoptosis and thus promote viral infection.

## Discussion

The domestic silkworm (*Bombyx mori*) is a key model organism for studying viral infections and immune responses. BmNPV infection significantly impacts the silkworm industry, and understanding its molecular mechanisms is critical for both silkworm protection and broader insights into viral infections in other lepidopteran insects [27]. In mammals, tetraspanins have been extensively studied in the context of viral invasion, where proteins like CD81 facilitate invasion by viruses such as chikungunya virus (CHIKV) and MERS-CoV [19,28]. Similarly, in arthropods, tetraspanins regulate viral entry, as seen with C189 in mosquitoes and TspanC8 in *Drosophila* [29,30]. Members of the tetraspanin superfamily play regulatory roles at various stages of viral infection, particularly during viral invasion, tetraspanins could coordinate interactions between virus and cell surface components [27]. By binding to other membrane proteins to form TEMs, which facilitate viral internalization through bridging the virus with the host cell [31]. A number of studies have demonstrated that TEMs can regulate the entry of a diverse range of viruses into cells, including those belonging to HPV [32], influenza A [31] and human immunodeficiency [33,34]. In this study, our findings reveal the pivotal role of *BmTsp.C* in promoting BmNPV proliferation, proving another basis for the study of tetraspanins in insect viral infection.

Many tetraspanins have been confirmed to contain palmitoylation sites, such as the tetraspanin CD9, which has been demonstrated to undergo palmitoylation at four internal juxtamembrane regions, with this palmitoylation contributing to the interaction between CD81 and CD53 [24], whereas palmitoylation of CD51 aids the stability of tetraspanins [35]. Therefore, we hypothesize that BmTsp.C possesses specific recognition capabilities in the viral infection process and binds to viral envelope proteins upon their accumulation.

Our experiments demonstrated that *BmTsp.C* is highly expressed in the midgut, which is involved in both digestion and immune defence [36]. The transcription level of *BmTsp.C* increased significantly at 48 h post-infection. This suggests that *BmTsp.C* may play an early role in recognizing and resisting the virus, while later facilitating viral replication by suppressing host defence mechanisms. Further experiments showed that *BmTsp.C* overexpression significantly enhanced BmNPV proliferation, as indicated by increased fluorescence intensity in BmN cells. Conversely, *BmTsp.C* knockdown resulted in reduced viral proliferation. These findings were confirmed by *in vivo* experiments, where *BmTsp.C* knockdown in silkworms led to healthier larvae, highlighting the protective role of *BmTsp.C*.

In the present era, overexpression and knockdown have assumed a pivotal role in elucidating the functional role of tetraspanins in regulating the expression of related genes. Our results provide a foundation for future studies exploring the mechanistic role of *BmTsp.C* in viral infection and apoptosis modulation, and the development of targeted strategies to suppress BmNPV infections. The results provide a foundation for future studies exploring the mechanistic role of *BmTsp.C* in viral infection and apoptosis modulation, and the development of targeted strategies to mitigate BmNPV infections.

One of the key findings in our study is the modulation of apoptosis signalling by *BmTsp.C*. Apoptosis is a primary defence mechanism against viral infection, with infected cells typically undergoing programmed cell death to prevent viral spread [37]. However, viruses often employ strategies to inhibit apoptosis to enhance their replication [38] . Our results showed that *BmTsp.C* overexpression in BmN cells resulted in the downregulation of pro-apoptotic genes such as *BmCaspase9* and *BmApaf1*, while anti-apoptotic genes such as *BmIAP* and *BmPI3K* were upregulated. This suggested that *BmTsp.C* plays a pivotal role in inhibiting apoptosis during BmNPV infection, which is consistent with findings in other viral infections, where tetraspanins regulate cell death pathways to favour viral replication [39].

Our study also highlights the PI3K-Akt signalling pathway as a critical mediator in *BmTsp.C*-induced apoptosis regulation. *BmTsp.C* overexpression led to the activation of this pathway, which subsequently suppressed caspase activation by upregulating BmIAP [40]. The PI3K-Akt signalling pathway leading to the activation of downstream *Bmp53* and transcriptional activation of *BmPTEN* [41], which resulted in the dephosphorylation of PIP3 to PIP2, while *BmPI3K* catalysed the phosphorylation of PIP2 to PIP3, further activating the *BmPI3K* signalling pathway and downstream regulatory pathways [42]. The pro-apoptotic gene *Bmp53* interacts with *BmBuffy* to promote its expression, thereby reducing cell apoptosis [43]. In the endogenous apoptosis pathway post-BmNPV induction, *BmcytC* is released from the mitochondria and binds with *BmApaf1* to form apoptotic bodies, which activate precursor caspase proteins, forming an apoptosis complex and triggering a cascade of apoptotic reactions [44]. Additionally, stimulation of the PI3K-Akt pathway promotes the expression of *BmBcl-2. BmBuffy*, a member of the *BmBcl-2* family, inhibits apoptosis by preventing the release of *BmcytC* and the activation of the Caspase family (Fig. S1) [45]. These findings indicated that *BmTsp.C* may regulate the apoptosis signalling pathway, inhibiting apoptosis induced by viral infection.

In conclusion, our study identifies *BmTsp.C* as a key player in BmNPV infection. By regulating apoptosis signalling pathways, *BmTsp.C* facilitates viral proliferation and survival. These findings lay the basis for future research aimed at understanding the mechanistic role of *BmTsp.C* in viral infection and highlight its potential as a target for controlling BmNPV spread.

## Supplementary material

10.1099/jgv.0.002098 Supplementary Material 1.

10.1099/jgv.0.002098 Supplementary Material 2.

## Abbreviations

BmNPV: *Bombyx mori* nucleopolyhedrovirus
CoV: Coronaviruses
cyt C: cytochrome C
qRT-PCR: quantitative real-time PCR
TEM: tetraspanin-enriched microdomain
TM: transmembrane
