# Supplementary material for: Functional analysis of BmTsp.C in modulating infection of BmNPV through apoptosis pathways in domestic silkworm (Bombyx mori)
Source: J Gen Virol. 2025 May 9;106(5):002098. doi: 10.1099/jgv.0.002098 (PMC12064854; doi:10.1099/jgv.0.002098)
Supplement: Supplementary Material 1. [file jgv-106-02098-s001.pdf]

Figure Legends

Figure S1. Figure S1. Protein topology diagram of BmTsp.C The diagram labels the large extracellular loop (LEL), small extracellular loop (SEL), N-terminus, and C-terminus. The four transmembrane regions of the TM are represented by numbers 1-4. The conserved amino acid residues are marked in *red*, disulfide bonds formed between amino acid residues are represented by *blue* lines, and the post-translational modification of the protein is shaded with *green*.

Figure S2. Multiple sequence comparison of BmTsp.C. Identical amino acid residues are indicated by asterisks (*). The purple box represents the amino acid sequence 'Cys-Cys-Gly', while the yellow box represents 'Pro-X-Ser-Cys'. The red amino acid letters indicate conserved cysteine residues, and the blue lines represent the disulfide bond between conserved amino acid residues. The solid lines on the sequence represent secondary structures, and the black bidirectional arrows TM1-4 indicate transmembrane structures. The sequence was downloaded from the NCBI database.

Figure S3. Phylogenetic tree of BmTsp.C. The branches with a yellow background contain BmTsp.C and only with bootstrap value over 70 are labelled. The scale of 0.10 represents a certain amount of genetic difference, and the same color represents the same species. The figure includes abbreviated names of different species. Bm: *Bombyx mori*; Bma: *Bombyx mandarina*; Ms: *Mythimna separata*; Tn: *Trichoplusia ni*; Lg: *Leguminivora glycinivorella*; Cp: *Cydia pomonella*; Of: *Ostrinia furnacalis*; Hz: *Helicoverpa zea*; Pa: *Pararge aegeria*; Mj: *Maniola jurtina*; Cs: *Chilo suppressalis*; Se:

Spodoptera exigua; Sf: *Spodoptera frugiperda*; Ls: *Leptidea sinapis*; Mc: *Melitaea cinxia*; Zc: *Zerene cesonia*; Pr: *Pieris rapae*; At: *Amyelois transitella*; Pn: *Pieris napi*; Pb: *Pieris brassicae*; Po: *Phthorimaea operculella*; Pb: *Pieris brassicae*; Vt: *Vanessa tameamea*; Cc: *Colias croceus*; Pa: *Pararge aegeria*; Ba: *Bicyclus anynana*; Ni: *Nymphalis io*; Va: *Vanessa atalanta*; Vc: *Vanessa cardui*; Pi: *Plodia interpunctella*; Pg: *Pectinophora gossypiella*; Ta: *Tuta absoluta*; Ms: *Mythimna separata*; Dpp: *Danaus plexippus*; Pb: *Pieris brassicae*; Aa: *Aricia agestis*; Se: *Spodoptera exigua*; Pi: *Plodia interpunctella*; Po: *Phthorimaea operculella*; Mh: *Maniola hyperantus*.

Figure S4. Apoptotic signaling pathway of *BmTsp.C* The vesicle membrane protein GP64 on BmNPV interacts with the tetraspanin BmTsp.C to promote apoptosis. Black arrows indicate promotion and green arrows indicate inhibition.

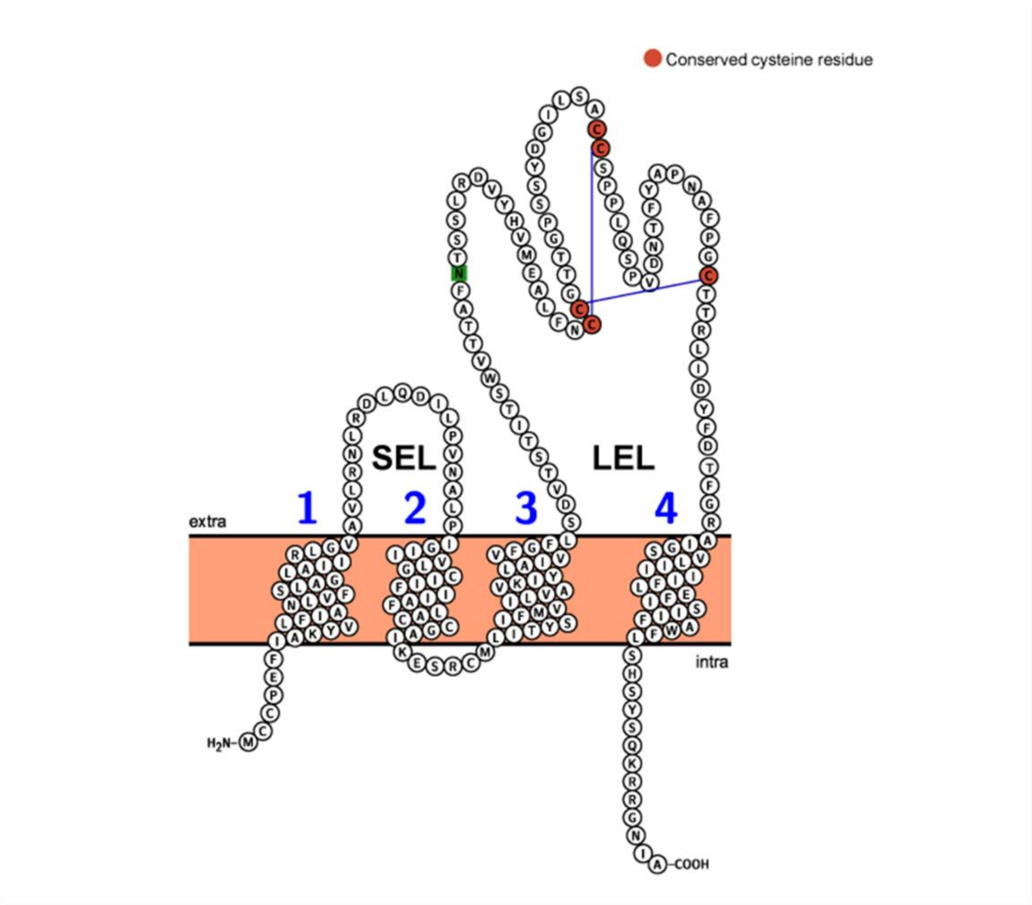

Figure S1. Protein topology diagram of BmTsp.C

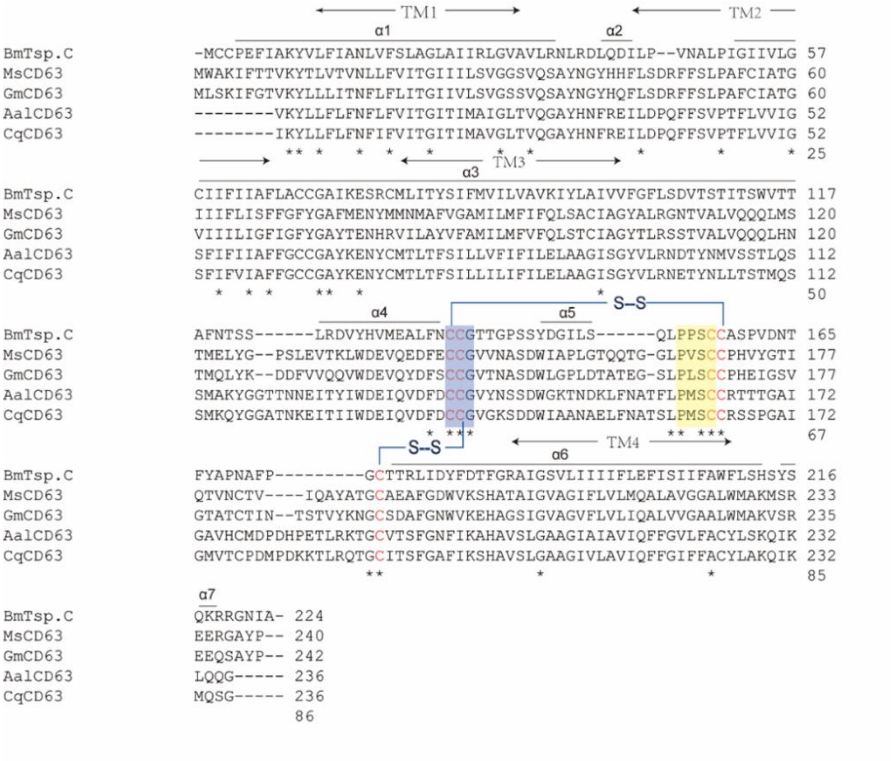

Figure S2. Multiple sequence comparison of BmTsp.C

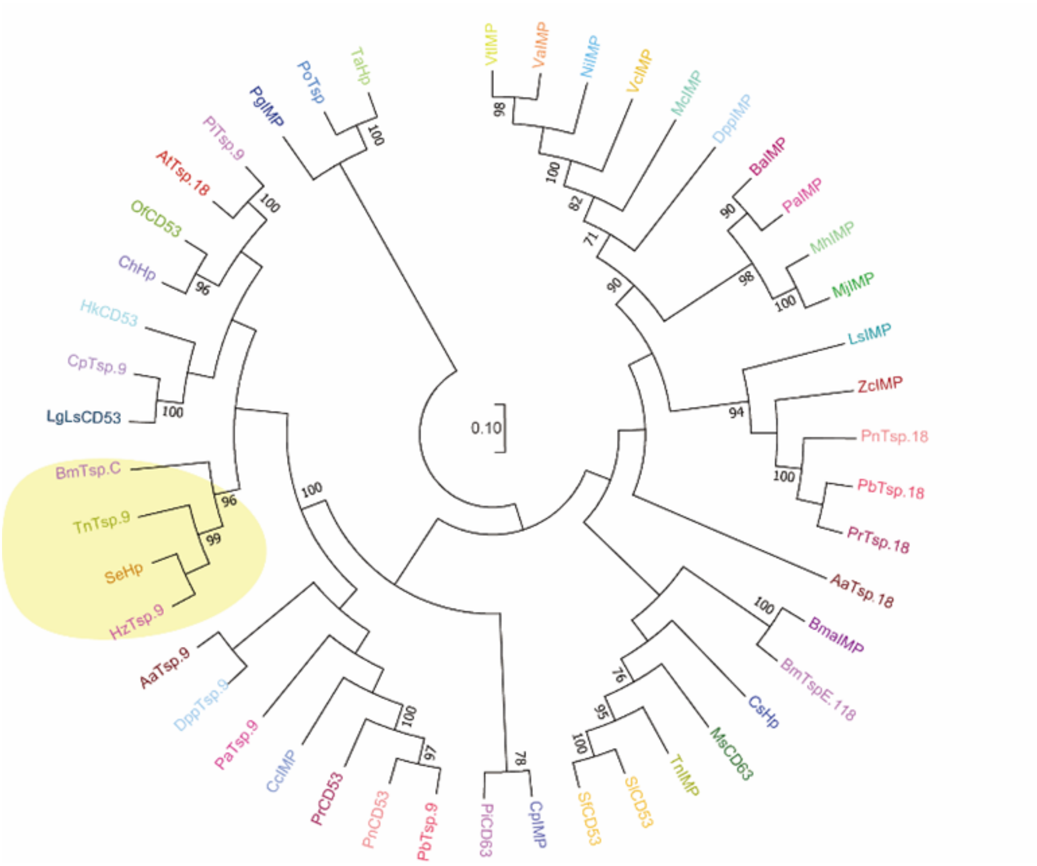

Figure S3. Phylogenetic tree of BmTsp.C

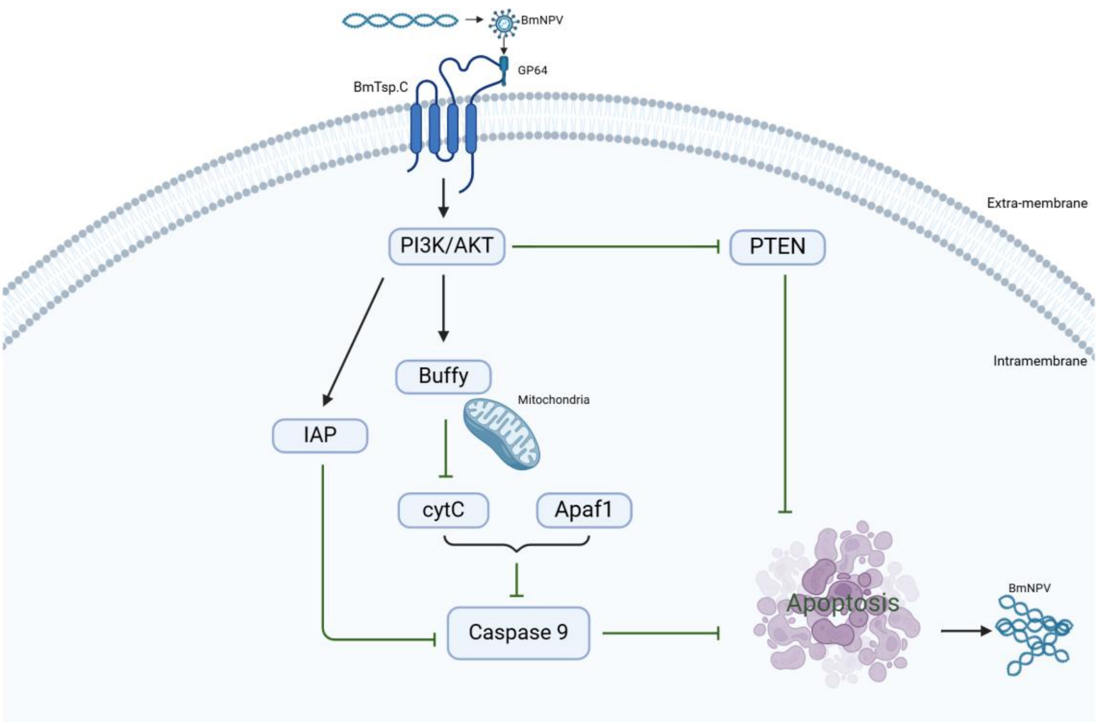

Figure S4. Apoptotic signaling pathway of *BmTsp.C*

Sequences of constructed phylogenetic tree

>BmTspC

MCCPEFIAKYVLFIANLVFSLAGLAIIRLGVAVLRNLRDLQDILPVNALPIGIIVL

GCIIFIIAFLACCGAIKESRCMLITYSIFMVILVAVKIYLAIVVFGFLSDVTSTITS

WVTTAFNTSSLRDVYHVMEALFNCCGTTGPSSYDGILSQLPPSCCASPVDNTF

YAPNAFPGCTTRLIDYFDTFGRAIGSVLIIIIFLEFISIIFAWFLSHSYSQKRRGNIA

-

>BmIMP

MCCPEFIAKYVLFIANLVFSDPQSRQFTSTDPNDEDLEPSILLSELAGLAIIRLG

VAVLRNLRDLQDILPVNALPIGIIV

LGCIIFIIAFLACCGAIKESRCMLITYSIFMVILVAVKIYLAIVVFGFLSDVTSTITS

WVTTAFNTSSLRDVYHVMEALF

NCCGTTGPSSYDGILSQLPPSCCASPVDNTFYAPNAFPGCTTRLIDYFDTFGRAI

GSVLIIIIFLEFISIIFAWFLSHSY

SQKRRGNIA

>BmCD53

MCCPEFIAKYVLFIANLVFSDPQSRQFTSTDPNDEDLEPSILLSELAGLAIIRLG

VAVLRNLRDLQDILPVNALPIGIIV

LGCIIFIIAFLACCGAIKESRCMLITYSIFMVILVAVKIYLAIVVFGFLSDVTSTITS

WVTTAFNTSSLRDVYHVMEALF

NCCGTTGPSSYDGILSQLPPSCCASPVDNTFYAPNAFPGCTTRLIDYFDTFARR

RQMSRASHRASQCTAKRTASSCGILH

RLSTICFRRSRLPRLTTHRNSHRSRLWPRVHLDHLRLVPVAFLFAETAREYSIRS

T

>MsHp

MCCLAEFIVKYVLIISNFIFAIAGLAIIGLGTAAQINLNEIHDVVPGSLSFLTISIIT

LGCIIFVIAFCACCGAIRESTC

MLLTYAVFMGILAAVKIYITVVIFEFLGHVTDTVTDWLTKAFANSNVRPAYNA

MENLFKCCGTVGPASYPTIGLPVSPTC

CKNPDMLVNECSLTNSYEGCIPQVTSYLESFGEAIGIVLIVVILVECVCLIFSIFL

VCQFRNKKRRYA

>TnTsp.9

MCCLEFIVKYVLIIGNIIFALLGLGILGLGIAVHVNLKDIYQLIPVGLSALSISIITL

GCIIFVISTLACCGAIRESRCM

LLFYALFMAILAGIKIYLTVLIFGFLDTATTTVTRWLISAFNNEDLRPAYHGLET

LFRCCGTTGAGSYAPGAVPQSCCAS

PIDNVCQANFYDGCIDQLTSYFETFGESIGAVLIVVIIIECVCVLAGLFLSCQIRR

KRYTA

>LgLsCD53

MCLAAFVVKYVLFFANAIFAIAGLALVGVGIAVLVQFGDLVEVVPAAINSIPIA

VLVVGAVVFLIAVFGCCGAIRESRCL

LIMYAVCMMILAAVKIYLAVVIFQNLSDIYNVVEGWIRDAFNNPNLREAFHVV

EAAFQCCGVSGPSSYDGVYPALPPTCC

ASADAGTCAAADAFGGCANIVAAWFNTFGDAVGIVLIIVIVVELVAMIFGLFLS

NRITNKKRAF

>HzTsp.9

MCCFAEFVVKYVLIIVNIVFCLAGLAIIGLGTAVHIQLDQIHELIPVNLSAISISII

VLGSFVFIISYCACCGALRESRC

MLIFYAVIMAILASLKIYITVVIFRFLDSALSTVSGWLNTAFNNMELRPAYYGM

ENLFRCCGTTGPNSYTDRGEVIPPSC

CGNLNNVTSNPENVQCAATDAFSGCTEMVGEYFETFGEAIGGVLIVVIIIELIC

VIFGLFLACQIRQKRYRA

>MjIMP

MFGYGTIKRRNTSRISVTLYVTLESIVIVNKPAIPYILPKTAKKPKMCFIVKYVL

FIANVIFALAGLTLIGVGSAVLALA

SDALAVIPAGINAIPISVIVLGAIVFVISFFGCCGAIRENRCFLTTYAIIMLLLAAG

KMYLAVVIFMAMDNIYVNVVEWL

TKAFLNEDLREPFHAVEIAFRCCGTTGWQSYLGILPALPASCCNPDAEVCQPD

NAFGGCNDIVGDFFATYGSATASILVV

IIAVELIAMIVGLSLCNNVSNRRRATV

>ChHp

MCCCAEFIIKYVLFFANFVFSLAGLALIGVGIAVLVQISGIDALPSGLNAIPISVI

VLGSIIFCIAFFGCCGAIRESQCL

LTMYFICMAILAAGKIYLATVIFNGLRTLDSIVTGWVNGAFDQSGRPGFRILEV

AFRCCGNTGPGAYAPDVLVPSSCCPD

PAADSDTCPVDDAFERGCTQAVSEYFETFGEAIGGVLITVIIIELVAMLFGMFLC

WSVRSNRR

>SeHp

MCCLAEFIVKYVLIIVNIVFALAGLAIIGLGTAVHVNLSDFYQIIDIRVLTISVIVI

GCIVFVIAFCACCGAIRESKCML

GFYAACMAILAAIKIYITVVIFSFLGHTMDTISRWLNTAFNNEDLRPVYQGME

MLFQCCGTNGTSSYLVDGRPIPASCCP

NPNAQPPQQCSTTNAYSGCVDKVSDYFDTFGEAIGGVLVVVIIVECICVIFGLF

MCCQIRGRRTV

>LsIMP

MGCGEFLVKYILFFANLFFALAGIALIGLGVAVQLQVSEVVNVIEGNFQVAPIT

AMVVGSIVFIIAFFGCCGAIRESNCM

LVTYSLFMIILMLIKVTLAVLIFVNLSDYTEGISQWLTNAFNKDRTAFQEIERTF

KCCGPQGAMSYLNVVLPETCCEGSP

CTVLNAYPSCSSTVEDFFQTFGVAIGSVAIVVAALELVAVVFALCLANHARNEG

RRSRY

>BaIMP

MGCGEFLVKYILFFANLFFALAGVALIGLGVAVELHVSKAADILQSSPIVQLTPI

GAIVVGCIVFVVAFFGCCGAIRESN

CMLITYAIFMIVLMVLKITLATLIFVKQDELLADIPRWLTDAFNKDRQAFQEIE

RTFTCCGVEGPQSYMSLALPATCCAA

GVQTCTVVNAHPGCSRVLSDFFQTFGLAIGVVAIVVVAVELVAVVFGLCLANH

VRNKSRRTRY

>MsHp

MGCGEFVVKYILFFANLVFALLGLALLGLGIAVQLKATDIVQIADFSLEVAPVT

SMVVGGIVFCIAFFGCCGAIRESNCM

LVTYAIFMLLLMAIKLTMAILIFVKLDNVVAAVPKWLTEAFQKDKIAFQAIEET

FSCCGPNGPQAYGQVVLPDTCCATPP

CVPFVNSYTGCDTHVQEFFQTFGIAIGSVMIVIVSIELVAAVFGLCLANTVRNK

GRRSRY

>DppTsp.9

MCCLLVKYGLFFSNLIFTLGGLALIGLGVATLLQLTDVLEVVTTNVNAIPITVIV

LGALIFLIAFLGCCGAIRENRCFLT

LYAICMIALAAAKLYVVVVVFRNLSDIKDLVKEWLEEAFLRLEIREQFYVMQS

TFQCCGTTGPNSYNVALPPSCCPSVVQ

TCEASSAFEGCNKVVADFFETYGEVIGIIVAVIVAIEVLAVVLSFSFCSTVGSNR

RRTV

>PxHp

MGCGEFIVKYVLFFANLFFALAGLALLGVGAAVQWQVKPLLDVFDGSFEVAP

ISAMVVGGVVFLIAFFGCCGSLRESNCM

LVTYAVFMIMLMILKITLASLIFVNLGAVTDQIPRWLNAAFNQPNLDEFHQIET

TFTCCGTVGGSSYVSLSLPPSCCTVQ

PCTVLNAHPGCNDVLTEFFRTFGMVIGLLAIAVVSVELVAVVFALCLANRARN

YQRRSRY

>SfHp

MGCGEFLVKYILFFANLVFALAGLTLLGLGIAVQLQSTDIVQIADYNIQIAPITS

MIVGGVVFFIAFFGCCGAIRESNCM

LVTYSIFMLLLMIIKLTLAILIFVKLDDVVSKVPLWLNEAFKQDQVSFQAIEKTF

TCCGPDGYSSYIGRDLSPTCCATPP

CISLTAYGGCNTHVQQFFQTFGLAIGSVMIVIVSLELVAAVFGLCLANTVRNKS

RRARY

>AtTsp.18

MGCGEFLVKYILFFTNLFFALAGLALLGVGIAVQLQVTVITNQIDVNLQLGPIT

TIVVGAVVFLVAFYGCCGAIRESNCM

LVTYSIFMIVLMIAKIALATFIFVNLSELTDEIKRHFTRLFQENQAAFQEIETAFS

CCGPLGAISYGTSILSLPDRCCAT

APCNISNAYGGCNDKVEGLMETYGMAIGVVAIVVVAVELVAVVFGLCLANHA

RNKDRRSLFITVLTDITEVLAGAGLVGV

GVAVLLHISDILDLIPVNLNAIPISIVVLGSIVFAIGFCGCYGAIKQSKCLLTMYA

VVMAVLAGVKIYLATILFRGLNNI

RETTEGWVVDGFGNRELFSALEFAFSCCGTTGPSSYGGITPVLPPTCCPSPTSD

GFCLPADAFQEGCVDRLASYFEVFGD

AIAAVIVVVITAELVALVFSLYLCCASKKR

>BmaIMP

MGCGEFLVKYVLFFANLAFALAGLALLGLGLAVHLQFADIETVLSETSFRVAP

VACMIVGGIAFIIAFFGCCGAIRESNC

MLVTYAIFMLVLMALKLTLGVMVFVNLDGVVAAIPNWMNKTFQQDQDTFHV

IENRFSCCGPTGPGSYLSLTLPDTCCSTT

PCTVINAYAGCTEVLQALFNNYGVAIGSVAIVIAAIELVAVIFALCLANHARNK

MRRSRY

>BmTspE.118

MGCGEFLVKYVLFFANLAFALAGLALLGLGLAVHLQFADIETVLSETSFRVAP

VACMIVGGIAFIIAFFGCCGAIRESNC

MLVTYAIFMLVLMALKLTLGVMVFVNLDGVVAAIPNWMNKTFQQDQDTFHV

IEHRFSCCGPTGPGSYLSLTLPNTCCSTT

PCTVINAYAGCTEVLQALFNNYGVAIGSVAIVIAAIELVAVIFALCLANHARNKI

EEVALLILV

>SlCD53

MGCGEFLVKYILFFANLVFALAGLTLLGLGIAVQLQSTDIVQIADYNIQIAPITS

MVVGGIVFFIAFFGCCGAIRESNCM

LVTYSIFMLLLMIIKLTLAILIFVKLDEVVAKVPFWLNESFKQDMVSFQAIEKTF

SCCGPEGASSYVRTPLSATCCATAP

CTISNAYSGCNEHVQQFFQTFGLAIGSVMIVIVSIELVAAVFALCLANTVRNKS

RRARY

>MhIMP

MGCGEFLVKYILFFANLFFALAGLALLGLGVAVELQVSQVAGVLEGSPVFKLT

PIGAIVLGCIVFVVAFFGCCGAIRESN

CMLITYAIFMIVLMVLKIALATLIFVKQDQLLDDIPTWLAEAFARDRQGFMEIQ

KSFTCCGVTGPESYLNVMLPESCCAG

TPCTIVNAHPGCNKVLTEFFKTFGLAIGIVAIIVVSVELVAVVFGLCLANHVRNR

SRRTRY

>HkCD53

MDTTASRGVGYDERKGLSLAPAPPPLSATIHRCRRRPPHYTPSRCRPCLAPRST

GIGRYSDLVGATFIGIGIAVLVALDK

ITDLIPAINTIPILVIVIGCIIFLIAFFGCCGAVRENGCLLIMYAVCMLIMAGANIW

LAVAIFNNLNGLMNTIEEWVDTA

FQQHGQTLNGMFRALEELLRCCGTTGKESYNDLLFIPSSCCPLENCDNADTY

YDGCSAKFTEYLTNFGNIVGYIVIVVIC

IEVTAMIFALFLNSRISKAKRGY

>HkIMP

MGIAVLVSLDRVAEIIPVITTIPILVIVVGCIVFLISFFGCCGAIRENGCLLIMYAVC

MFLLVASNIALAVVIFRNLNGL

STTVDGWVRTAFQQHGQAPDGGFRALEELFKCCGTTGKESYNNLPFIPPTCCP

EVNCDSAATYYDGCSTIFTDYLNNFGN

VVGTIVIIILCIEFIAMIFALFLNQQITSAKRRQRY

>DppIMP

MGCGEFLVKYVLFFANLFFALAGLALLGLGIAVELRISNAAEILDKSPIFQLTPI

GAMVIGSIIFLVAFFGCCGAIRESN

CMLIVYSIFMIMLMILKVTLATLIFVKQDELLTNIPVWLNETFTRDQVAFQGIE

KSFSCCGPAGAASYGLAPLPATCCAN

TPCTLINAYSGCGDIVTEFFETFGLAIGSVAIVVGAIELVAVVFSLCLANHARNK

NRRSRY

>PbTsp.9

MCFIVKYVLLIANFIFTLAGLTLLGLGIAALVNTSDLSGVATTPLNAIAISIIVLG

ALVFLIAFFGCCGSMKENRCLLLT

YALCLLLLAGAKIYLTVLMFTSEDSVGDLVEEWIEKAFYNNELRDTMHAIETA

FECCGTTGPNSYFGILQALPPSCCPYT

VSQRCEISNAFGGCINIITEFLSKYSDVFGGILASILAIEFVAMLFAVMLACNIGA

KRR

>CpIMP

MGCGEFLVKYILFFANLFFALSGLALLGIGVAVQLKVTAITSLIDGWYEFGPIAT

MVVGSIVFFIAFLGCCGAIRESNCM

LVTYSIFMIVLMVLKIALATIVFVNADSLATSIKNEFNNVFLNNQTEFHKIETAL

TCCGTEGGQSYGVNIVSLPSSCCAE

GAAACTLLNSYPGCNSRAEDFINTFSTAIGAVCIVVAALELVAVVFGLCLANHV

RNRDRRTRY

>AaTsp.18

MGCGEFLVKYILFFANLFFALAGLALLGIGIAVELKVAAVVNFMEQNQMLQM

TSISAIVLGSVVFVIAFFGCCGAVRESN

CMLVTYSIFMLVLMVLKITLAVLIFVNLDSFLAEVPKWLNEAFAKDQQAFHEL

EQSLKCCGPTGASSYQNLVLPQSCCAS

TPCTIVNAYTSCNDVIQSFFSTFGLVTGAVAIGIVAVELVAVVFGLCLANHVRNR

DRRAYY

>SfCD53

MGCGEFLVKYILFFANLVFALAGLTLLGLGIAVQLQSTDIVQIADYNIQIAPITS

MIVGGIVFFIAFFGCCGAIRESNCM

LVTYSIFMLLLMIIKLTLAILIFVKLDDVVSKVPLWLNEAFKQDQVSFQAIEKTF

TCCGPDGYSSYVGRDLSPTCCATPP

CTSLTAYGGCNTHVQQFFQTFGLAIGSVMIVIVSLELVAAVFGLCLANTVRNKS

RRARY

>HaIMP

MGCGEFLVKYILFFANLVFALAGLTLLGLGVAVQLQSSDIVQIADFNFEVAPITS

MVVGGIVFFIAFFGCCGAIRESNCM

LVTYSIFMLLLMIVKLTLAILIFVKLDDVVNEVPKWLKEAFNKDRTEFQAIERT

FTCCGPDGALSYMSPLLPDTCCATPP

CTPVNPYPSCTQNVQEFFQTFGVAIGSIMIVIVSIELVAAVFGLCLANTVRNKSR

RAHY

>AaTsp.9

MCCIMKYGLFVTNLVFSLAGLGLLGLGIAVVVQVTELTDLVPAGITTIPITLLVV

GGFIFVTALLGCCGAIRENRCFLML

YAIIIFVLAAGKTYVAVATWRAVSSIRESVVEWLGDAFANPEMREPFHVMETAF

RCCGTTGAAAYEGPLPVSCCPDDVAC

GLATAYGGCNDRIGTFAETYGQAIGAVVIIVIAVELILAAFTICFCTSITKSSTAK

V

>SfHp

MGCGEFLVKYILFFANLVFALAGLTLLGLGIAVQLQSTDIVQIADYNIQIAPITS

MIVGGVVFFIAFFGCCGAIRESNCM

LVTYSIFMLLLMIIKLTLAILIFVKLDDVVSKVPLWLNEAFKQDQVSFQAIEKTF

TCCGPDGYSSYIGRDLSPTCCATPP

CISLTAYGGCNTHVQQFFQTFGLAIGSVMIVIVSLELVAAVFGLCLANTVRNKS

RRARY

>MlHp

MGCGEFVVKYILFFANLVFAILGLALLGLGIAVQLKATHIVQIADFSLEVAPVTS

MVVGGIVFFIAFFGCCGAIRESNCM

LVTYAIFMLLLMAIKLTMAILIFVKLDNVVAEVPKWLNEAFQKDRVAFQAIEQ

TFSCCGPSGPAAYGPVALPDSCCATSP

CVILVNAYPGCEGHVQEFFQTFGIAIGSVMIVIVSIELVAAVFGLCLANTVRNK

GRRSRY

>CsHp

MGCGEFLVKYILFFANLFFALAGLTLLALGVVVQLKIADLSEMGHGQIQIAPIS

AMVVGGVVFLIAFFGCCGAIRESNCM

LVTYSIFMLILMVAMIALATAIFVSLEKVLSQVPEWLTQSFKTDENGFHAIETTF

HCCGPTGPSSYLSPVLPNSCCAQAP

CVPVVNAFPGCDTVVKEFLNTFGLVIGLVAIAVAAIQLVAAVFGLCLANHARH

KYRRSYY

>PiCD63

MGCGEFLVKYILFFTNLFFALAGLALLGVGIALQLNVTAITNQIDVNLQLGPIT

TIVVGAIVFLVAFYGCCGAIRESNCM

LVTYSISMIVLMITKIALATVIFVNLPQVIDEIKRHITQLFQDKPSAFHEIETAFSC

CGPSGPVSYGSPFLTLPDTCCAT

PPCNEGNAYGGCNARVEAVMHTYGLAIGVVAIVVVSIELVAVVFGLCLANHA

RNKARRSRY

>PoTsp

MGCGEFIVKYVLFFSNLFFSLSGLALVGVGIAVQLKVSAVTDILQDYNLAIAPV

SAMVVGGVVFLIAFFGCCGAIKESNC

MLVTYSIFMLVLMALKIALATLIFVNLGSLLQEIPKIMNESFAKDQKTFQQTVE

YTFSCCGPNGPLSYGVLLTLPDTCCA

VPTCTVGNAYGGCSDKVIDFTSTFGNLIALGAIVIGAIEVSNTVNLITCIIIGEVY

P

>BaIMP

MILAGVALIGLGVAVELHVSKAADILQSSPIVQLTPIGAIVVGCIVFVVAFFGCC

GAIRESNCMLITYAIFMIVLMVLKI

TLATLIFVKQDELLADIPRWLTDAFNKDRQAFQEIERTFTCCGVEGPQSYMSL

ALPATCCAAGVQTCTVVNAHPGCSRVL

SDFFQTFGLAIGVVAIVVVAVELVAVVFGLCLANHVRNKSRRTRY

>TnIMP

MGCGEFLVKYILFFANLVFALAGLALLGIGVAVQLQISQIINVIENADASLQTAP

ITSMVVGGIVFLIAFFGCCGAIREN

NCMLVTYSIFMLILMIIKLTLAILIFVKLDDIIAEVPQWLNEAFSRDKVSFQEIEK

TFSCCGPNGSLSYLSMNLPESCCA

TTPCTPINAYSGCNGTVQEFFHTFGLAIGVVAIVVVAIELVAAVFGLCLANHVR

NKGRRARY

>BmTspE.118

MGCGEFLVKYVLFFANLAFALAGLALLGLGLAVHLQFADIETVLSETSFRVAP

VACMIVGGIAFIIAFFGCCGAIRESNC

MLVTYAIFMLVLMALKLTLGVMVFVNLDGVVAAIPNWMNKTFQQDQDTFHV

IEHRFSCCGPTGPGSYLSLTLPNTCCSTT

PCTVINAYAGCTEVLQALFNNYGVAIGSVAIVIAAIELVAVIFALCLANHARNKI

EEVALLILV

>MsHp

MGCGEFLVKYILFIANLVFALAGLGLLGIGIAVQLQISAITNLITDSSFQVAPISS

MVVGGIVFVVAFFGCCGAIRESNC

MLVTYSIFMLVLMVLKLTLAVLIFVKLDDVVQEIPRWLNEVFNRDPIGFHEIER

TFQCCGPAGPVSYLNLKLPDTCCASP

PCNIVSSDNFSGCNGILQDFFNTFGLVIGVVAIVVVAIELVAVVFGLCLANHVRN

KSRRARY

>SlCD63

MGCGEFLVKYILFFANLVFALAGLTLLGLGIAVQLQSTDIVQIADYNIQIAPITS

MVVGGIVFFIAFFGCCGAIRESNCM

LVTYSIFMLLLMIIKLTLAILIFVKLDEVVAKVPFWLNESFKQDMVSFQAIEKTF

SCCGPEGASSYVRTPLSATCCATAP

CTISNAYSGCNEHVQQFFQTFGLAIGSVMIVIVSIELVAAVFALCLANTVRNKS

RRARY

>CcIMP

MGLLAKYVLFCANSIFTLAGLTLIGLGTAVVVHTAELMVLMPIAIEAIPISVIIIG

ILVLIISCIGYCGSIHENRFLLLV

YSLSTVLLAGGKIYLAIVIYNSVNRMPELVLDWLGQAFNNNQMRESFHAMEA

AFSCCGTTGPDSYTTGVLPSLPATCCPD

DIEDVCNSTNAFDGCYRIVTVYLESYGLVLGGLMVMIIIVELSASLFGMILCC

WIGNKRRRSV

>MjIMP

MGCGEFLVKYILFFANLFFALAGLALLGLGVAVQLQVAQVEDVLKGSPVFQLT

PIGAIVLGCIVFVVAFFGCCGAIRESN

CMLITYAIFMIVLMVLKIALATLIFVKQDQLLDDIPTWLAEAFAKDRQGFMEIQ

QSFSCCGVTGPESYLNVMLPESCCAG

TPCTIVNAHPGCNKVLSEFFKTFGLAIGVVAIVVVAVELVAVVFGLCLANHVRN

KSRRTRY

>MsCD63

MGCGEFLVKYILFIANLVFALAGLGLLGIGIAVQLQISAITNLITDSSFQVAPISS

MVVGGIVFVVAFFGCCGAIRESNC

MLVTYSIFMLVLMVLKLTLAVLIFVKLDDVVQEIPRWLNEVFNRDPIGFHEIER

TFQCCGPAGPVSYLNLKLPDTCCASX

PCNIVSSDNFSGCNGILQDFFNTFGLVIGVVAIVVVAIELVAVVFGLCLANHVRN

KSRRARY

>MhIMP

MGCGEFLVKYILFFANLFFALAGLALLGLGVAVELQVSQVAGVLEGSPVFKLT

PIGAIVLGCIVFVVAFFGCCGAIRESN

CMLITYAIFMIVLMVLKIALATLIFVKQDQLLDDIPTWLAEAFARDRQGFMEIQ

KSFTCCGVTGPESYLNVMLPESCCAG

TPCTIVNAHPGCNKVLTEFFKTFGLAIGIVAIIVVSVELVAVVFGLCLANHVRNR

SRRTRY
